# Plica Neuropathica (Plica polonica) Following Azathioprine-induced Pancytopenia

**DOI:** 10.4103/0974-7753.77523

**Published:** 2010

**Authors:** Rajiv Joshi, Simran Singh

**Affiliations:** P. D. Hinduja Hospital, Mahim, Mumbai, India

**Keywords:** Azathioprine, pancytopenia, plica neuropathica, plica polonica

## Abstract

A 54-yr-old woman, on azathioprine for interstitial lung disease, developed pancytopenia and presented with sudden onset of extensive hair loss from the scalp followed overnight by appearance of elongated broad mass of uncombable matted hair which had the typical appearance of Plica neuropathica. Microscopic examination of hair clipped from the matted mass revealed irregular, nodal, superficial fractures of the hair shaft resembling Trichorrhexis nodosa and irregular ruffling of the cuticles. The areas of cuticular damage appeared dark under polarized light. Plica neuropathica following pancytopenia is unusual and the findings of hair shaft cuticular damage suggests that changes in surface characteristics of hair shafts may have led to the irreversible matting leading to development of plica along with extensive hair loss due to anagen effluvium.

## INTRODUCTION

Plica neuropathica is an uncommon condition that occurs due to sudden and complete matting of scalp hair leading to the formation of elongated stiff mass of hair that looks similar to dreadlocks.

It has historically been known as Plica polonica or the Polish plait[1] and usually results from neglect of hair care. Neglected and uncombed hair becomes irreversibly tangled and forms a matted, malodorous, moist stiff mass of hair. Patients often have pediculosis capitis and associated scalp inflammation. Plica polonica presents typically as an elongated firm to hard impenetrable mass of keratin permanently cemented together with crusted pus, blood, nits and dirt.[1]

The exact mechanism for matting is not well-understood but both physical and chemical factors that damage hair shafts may play a role.

The process of matting is essentially similar to the phenomenon of ‘felting’ which occurs in wool and textile industries in which there is compacting of contiguous fibers exposed to surface damage and friction.[2] Other contributory factors in pathogenesis of this condition are long hair, longitudinal splitting and weathering of hair, vigorous rubbing of hair in a rotatory manner and frequent use of harsh shampoos and hair cleansers.[3,4]

The other mechanism of development of Plica is neglect of hair care which may result in severe infestations with resultant exudates causing matting of hair.

Psychiatric morbidities may also result in development of Plica due to neglect of hair, secondary infestations and religious or superstitious beliefs and a case of Plica neuropathica has been described in a psychiatrically ill patient.[5]

Plica neuropathica generally affects healthy persons but has been described in a 14-yr-old chronically ill girl with azathioprine-induced pancytopenia, in the absence of shampooing or prolonged neglect of hair.[6]

Our case is to the best of our knowledge the first Indian case with Plica neuropathica following pancytopenia due to azathioprine and the first reported with hair shaft cuticular damage that resembles Trichorrhexis nodosa.

## CASE REPORT

A dermatological consult was requested for a 54-yr-old woman for complaints of sudden severe loss of scalp hair with matting of hair leading to elongated uncombable mass of matted hair.

She is a known diabetic on regular treatment, and was admitted to hospital for fever, oral ulcerations and pancytopenia. She had been started on prednisolone 10 mg daily and azathioprine 50 mg daily for her interstitial lung disease in early August 2010. Almost a month later in early September 2010, she developed fever followed 4-5 days later by oral ulcers and severe fatigue and was diagnosed to have pancytopenia with a markedly reduced WBC count of 1000, N-15%, L-76%, E-1%, M-8%, Hb of 9.3 g and platelets 1.1 lakhs.

On questioning, a few days before admission to hospital she developed sudden extensive loss of scalp hair that came out in bunches mainly from the front of the scalp. On attempting to comb her hair the remaining hair got matted together to form an elongated stiff protruberent mass extending downward from the back of the top of her head.

There was no associated pruritus or pain of the scalp.

She denied history of lice and used to wash her scalp once a week with ordinary shampoo. There was no history of use of any chemical treatment for her hair like streaking or straightening of hair in the past.

**Figure 1:**
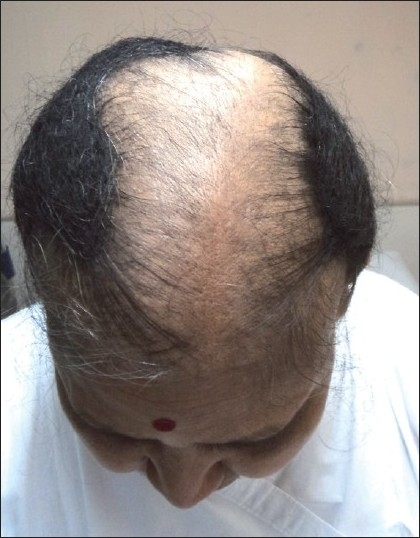
Extensive non-cicatricial alopecia involving anterior two-third of scalp

**Figure 2:**
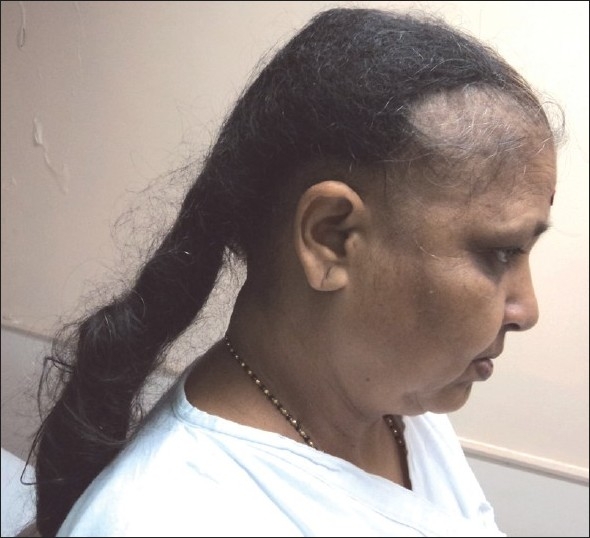
Elongated mass of stiff tangled hair (Plica)

**Figure 3:**
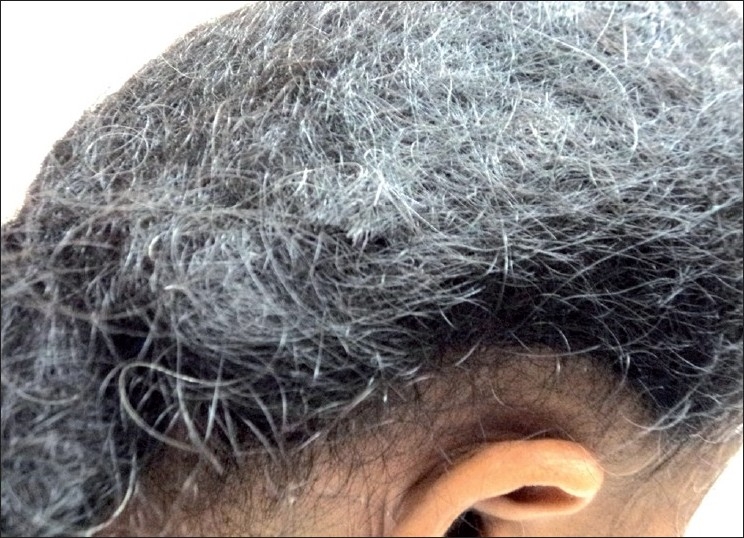
Close up of tangled hair

On examination she had extensive non-cicatricial alopecia involving the middle two-third of the scalp extending from the frontal hair margin upto the vertex [Figure 1], an elongated stiff mass of tangled hair extending downwards from the occipital region [Figure 2], which on closer examination was seen to be made up of tangled matted hair which were rough in appearance but without any adherent nits, lice or crusts [Figure 3].

Pull test of hair showed few hairs with anagen roots.

**Figure 4:**
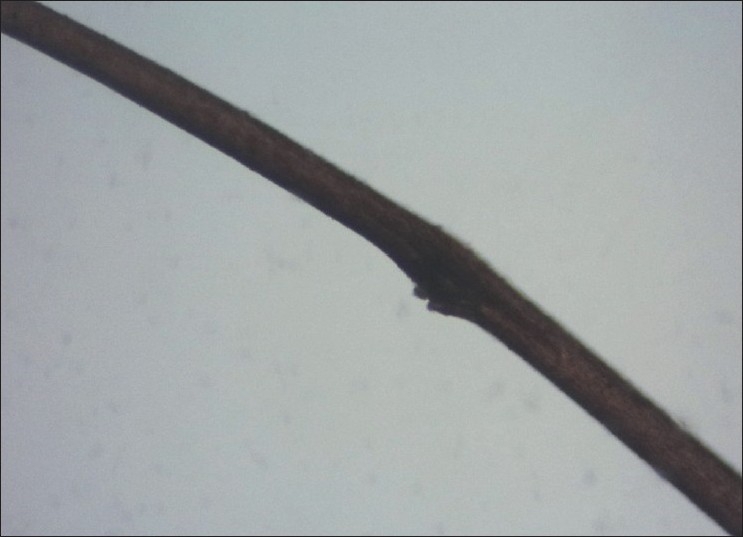
Focal break in surface cuticle resembling Trichorrhexis nodosa

**Figure 5:**
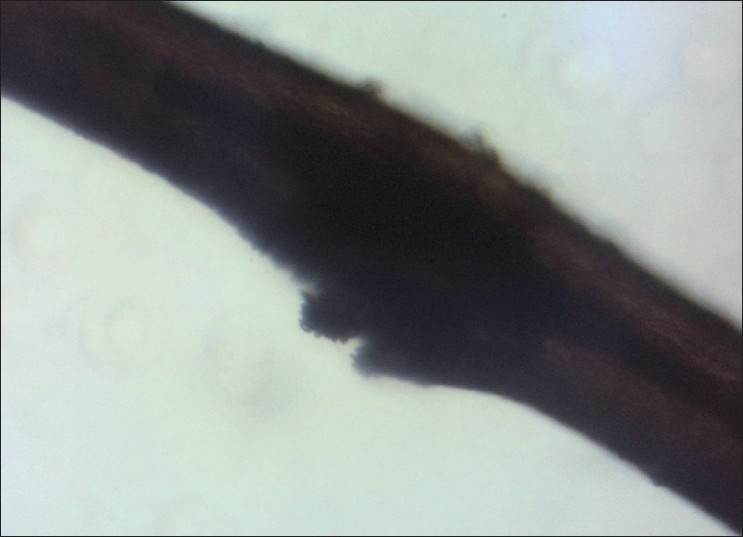
Higher power of the hair shaft damage

**Figure 6:**
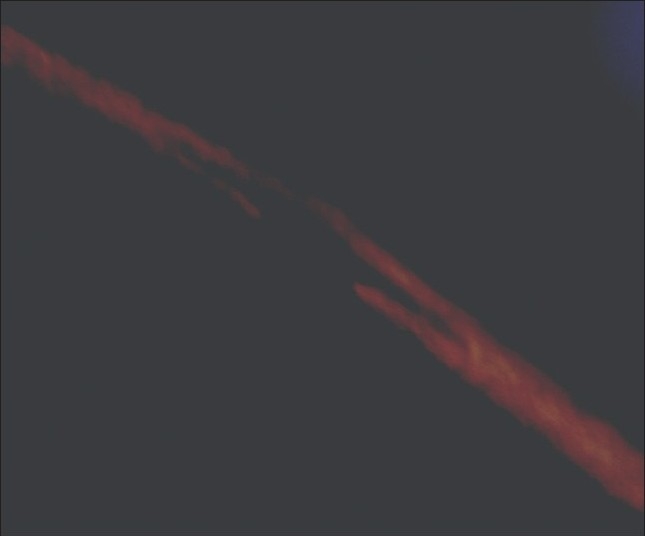
Dark empty appearance of damaged area of hair shaft under polarized light with the rest of the shaft appearing bright against a black background

Examination of hair clipped from the Plica revealed hair shafts which showed irregular, nodal, superficial fractures of the hair shaft resembling Trichorrhexis nodosa, with irregular fractures and ruffling of the hair cuticle [Figures 4 and 5] and a dark empty appearance of these areas of cuticular damage under polarized light [Figure 6].

Her stay in hospital was uneventful. Azathioprine was stopped and she was managed with supportive measures and her counts gradually came back to near normal within a week and she was discharged from the hospital.

Her hair condition remained the same, she was explained the nature of her hair loss and advised to get the plicated mass of hair cut as there was no effective treatment to untangle the adherent mass.

## DISCUSSION

Our case is similar in presentation to a reported case of Plica neuropathica in a 14-yr-old girl who had multiple chronic illnesses and who developed sudden hair loss and Plica while in hospital for treatment of pancytopenia due to azathioprine.[5]

The sudden and extensive hair loss from the anterior two-third of the scalp in our patient may have been due to anagen effluvium secondary to azathioprine toxicity and subsequent cuticular damage.

In anagen effluvium, commonly induced by anticancer drugs, hair loss is induced by an abrupt cessation of mitotic activity in the rapidly dividing hair matrix cells so that either no hair is produced, or a narrowed and defective hair shaft is produced and the hair loss is evident within days to weeks of drug administration.[7]

However, azathioprine is not normally known to be a cause of anagen effluvium and it is possible that the sudden pancytopenia experienced by the patient resulted in hair shaft damage leading to hair loss and hair shaft cuticular damage resulting in development of Plica.

The findings of hair shaft cuticular damage resembling Trichorrhexis nodosa has not been described before. The cuticular damage seen under the microscope as ragged irregular breaks in the cuticle may have predisposed to matting of the hair that resulted in development of Plica.

In conclusion, we describe a case of Plica neuropathica that developed following sudden extensive scalp hair loss associated with azathioprine-induced pancytopenia.

This is probably only the second case of Plica neuropathica associated with azathioprine-induced pancytopenia in literature and unusual in that it occurred not because of neglect and poor hair care or overzealous shampooing of hair but due to cuticular damage of hair shaft secondary to the azathioprine-induced pancytopenia and alopecia.
